# Effectiveness of acupuncture for anxiety and depression in irritable bowel syndrome: A protocol for systematic review and meta-analysis

**DOI:** 10.1097/MD.0000000000024958

**Published:** 2021-02-26

**Authors:** Huaiyu Li, Yun Chen, Ziyi Hu, Jiawang Jiang, Jing Ye, Yuliang Zhou, Zhiying Yu, Haiyi Tang

**Affiliations:** a Jiangxi University of Traditional Chinese Medicine, Nanchang; b First Affiliated Hospital of Gannan Medical University, Ganzhou; c The Affiliated Hospital of Jiangxi University of Traditional Chinese Medicine, Nanchang, P.R. China.

**Keywords:** acupuncture, anxiety, complementary therapy, depression, irritable bowel syndrome

## Abstract

**Background:**

Irritable bowel syndrome (IBS) is the most commonly gastrointestinal diseases. The Rome Foundation's global study on 33 countries shows the total prevalence of IBS under the Rome IV Diagnostic Criteria was 3.8%. It is well established that people with IBS have higher levels of anxiety and depression. The impact of the acupuncture associated with anxiety and depression has been widely studied in Western countries. Acupuncture may be a promising choice for the treatment of anxiety and depression in IBS.

**Methods:**

RCTs of acupuncture for depression and anxiety in IBS will be searched in the relevant database, including PubMed, Embase, Cochrane Library, China National Knowledge Infrastructure (CNKI), Wanfang Database, Chinese Biomedical Literature Database (CBM), and Chinese Scientific Journal Database (VIP database). The studies of electronic searches will be exported to EndNote V.9.1 software. We will run meta-analyses using the Review Manager (RevMan) V.5.3 software. Any disagreement will be solved in consultation with a third reviewer.

**Results:**

Our study aims to explore the efficacy of acupuncture for depression and anxiety in IBS and to provide up-to-date evidence for clinical of IBS.

**Conclusion:**

This study will perform a comprehensive systematic review and meta-analysis on the efficacy of acupuncture for depression and anxiety in IBS, making up for the lack of relevant evidence of the clinical use of acupuncture.

**INPLASY registration number:**

INPLASY 202120014.

## 1 Introduction

Irritable bowel syndrome (IBS) is one of the most commonly gastrointestinal diseases, and it is characterized by recurrent abdominal pain, discomfort, and altered bowel habits without any other organic disease.^[1]^ According to patients’ bowel habits, IBS is classified into 4 main subtypes: IBS with predominant constipation (IBS-C), IBS with predominant diarrhea (IBS-D), IBS with mixed bowel habits (IBS-M), and IBS unclassified (IBS-U).^[2]^ The Rome Foundation's global study on 33 countries shows that the total prevalence of IBS under the Rome IV Diagnostic Criteria was 3.8% (3.6, 4.0), and the prevalence of IBS-C among women is higher than that of IBS-D, while among men this is on the contrary.^[3]^ IBS has caused heavy pressure on individual life, health care and social economy.^[4]^

The pathological mechanism of IBS is complicated. It is well established that the pathogenesis of IBS is mainly related to altered visceral sensitivity,^[5]^ brain-gut axis dysfunction,^[6]^ intestinal dysfunction,^[7]^ somatic and psychiatric comorbidities,^[8]^ intestinal microbial imbalance,^[9,10]^ and other aspects. The diversity of pathogenesis makes IBS often have other non-gastrointestinal symptoms. Many evidences indicate that most patients with IBS often have more serious psychosocial problems,^[11–14]^ of which anxiety and depression are the most common.^[15–17]^ These psychological disorders accompanied by IBS are closely related to the two-way regulation between the central and enteric nervous system.^[18]^ Long-term psychological barriers not only cause IBS patients to suffer physical and mental torture, but also face many hidden dangers such as weakening of the social labor force, reduced treatment compliance, and increased suicide risk.^[19,20]^ For IBS anxiety and depression, small doses of antidepressants and psychological treatment play a certain therapeutic role. However, due to the side effects of antidepressants, these drugs are not licensed to treat patients with IBS anywhere in the world^[21]^; in contrast, in many Asian and developing countries, psychotherapy has not been effectively developed. The actual benefits of these two treatments are relatively limited.

As a traditional therapy, acupuncture has been widely used in various western medicine conditions, and it may also be a promising choice for the treatment of anxiety and depression in IBS. In recent years, a large number of studies have shown that acupuncture is effective for various types of depressive disorders.^[22–25]^ Although there are some studies on acupuncture treatment of IBS anxiety and depression, the meta-analysis of acupuncture treatment of IBS anxiety and depression is still relatively vacant. Therefore, we will conduct a meta-analysis using data from all relevant randomised controlled trials (RCTs) to comprehensively evaluate the efficacy of acupuncture in the treatment of anxiety and depression of IBS.

## 2 Objectives

The aims are:

1. to explore the efficacy of acupuncture for depression and anxiety in IBS and
2. to provide up-to-date evidence for clinical of IBS.

## 3 Methods and analysis

### 3.1 Study registration

The protocol of our study is conducted in strict accordance with the PRISMA-P guidelines and the Cochrane Handbook.^[26,27]^ This protocol has been registered on INPLASY (registration number: INPLASY 202120014: https://inplasy.com/inplasy-2021-2-0014/).

### 3.2 Inclusion criteria

#### 3.2.1 Type of studies

All RCTs which compared acupuncture with either placebo or other drugs. RCTs conducted in adults (participants aged >16 years) without regional and language restrictions.

#### 3.2.2 Type of participants

All patients with IBS, regardless the gender, age, race, country, and IBS type. Diagnosis of IBS based on specific diagnostic criteria (Rome I criteria, Rome II criteria, Rome III criteria, Rome IV criteria or the Manning criteria).

#### 3.2.3 Type of interventions

The experimental group is defined as acupuncture treatment, such as body acupuncture, warm acupuncture, electro-acupuncture, auricular acupuncture, fire needling, elongated needle, or moxibustion.

#### 3.2.4 Type of comparators

The control group that will include non-acupuncture techniques, such as sham acupuncture, placebo, adjuvant chemotherapy or other pharmacotherapy. The acupoint numbers, retaining time, and frequency will not be restricted in this protocol.

#### 3.2.5 Types of outcome measures

##### 3.2.5.1 Primary outcomes

The primary outcomes assessed will be the Hamilton Anxiety (HAMA) scale.

##### 3.2.5.2 Secondary outcomes

Secondary outcome measures include the Self-Rating Depression Scale (SDS), the Self-Rating Anxiety Scale (SAS), the Hamilton Depression (HAMD) scale, and the rate of adverse effects (AEs).

### 3.3 Exclusion criteria

- Non-RCTs;
- None of the valid outcome indicators;
- Duplicated literature;
- The data used for synthesis are incomplete;
- Animal studies, case-controlled studies, cohort studies, and case reports.

### 3.4 Search methods for identification of studies

#### 3.4.1 Electronic searches

RCTs of acupuncture for depression and anxiety in IBS will be searched in the relevant database, including PubMed, Embase, Cochrane Library, China National Knowledge Infrastructure (CNKI), Wanfang Database, Chinese Biomedical Literature Database (CBM), and Chinese Scientific Journal Database (VIP database). The key words include “acupuncture,” “electro-acupuncture,” “warm acupuncture,” “irritable bowel syndrome,” “IBS,” “depression,” “anxiety.” An equivalent translation of the same search terms will be used to search in the Chinese databases. The search strategy of PubMed is shown in Table 1.

**Table 1:** Search strategy used in PubMed database.

Order	Search items
#1	(((((((((Irritable bowel syndrome) OR (Irritable Bowel Syndromes)) OR (Syndrome, Irritable Bowel)) OR (Syndromes, Irritable Bowel)) OR (Colon, Irritable)) OR (Irritable Colon)) OR (Colitis, Mucous)) OR (Colitides, Mucous)) OR (Mucous Colitides)) OR (Mucous Colitis) [All Fields]
#2	((((((((((((((((Acupuncture) OR (acupuncture therapy)) OR (Electroacupuncture)) OR (electroacupuncture therapy)) OR (manual acupuncture)) OR (moxibustion)) OR (Acupuncture, Ear)) OR (Acupunctures, Ear)) OR (Ear Acupunctures)) OR (Auricular Acupuncture)) OR (Ear Acupuncture)) OR (Acupuncture, Auricular)) OR (Acupunctures, Auricular)) OR (Auricular Acupunctures)) OR (warm acupuncture)) OR (fire needling)) OR (elongated needle) [All Fields]
#3	randomized controlled trial[Publication Type] OR randomized[Title/Abstract] OR placebo[Title/Abstract]
#4	#1 AND #2 AND #3

#### 3.4.2 Searching other resources

We will search the National Institutes of Health (NIH) clinical registry Clinical Trials, International Clinical Trials Registry Platform (ICTRP), and ClinicalTrials.gov to find the any potentially eligible trial data.

### 3.5 Selection of studies

**Figure 1:**
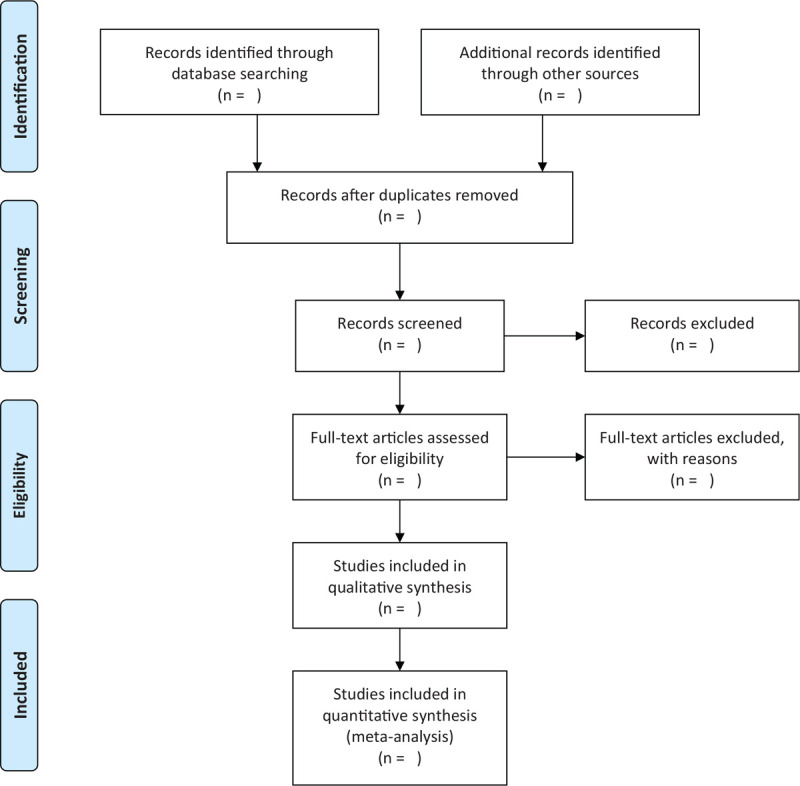
Flowchart of literature selection.

The studies of electronic searches will be exported to EndNote V.9.1 software. Two authors will independently undertake the process of selecting the search results according to the inclusion and exclusion criteria. They will review and screen the titles and abstracts retrieved by literature search to exclude irrelevant trials. The causes of both selections will be documented and full texts will be obtained and checked for further evaluation if necessary. When there is uncertainty about eligibility of the study, reviewers will arrive at a decision by via discussion and consensus with a third reviewer. The selection process will be showed in a PRISMA flow diagram (Fig. 1).

### 3.6 Data extraction and management

Data will be extracted independently from the selected articles by two reviewers using a Microsoft Excel spreadsheet. Information extracted from each included article will include first author, publication time, study types, characteristics of participants, type of treatments, outcome measures, and adverse events. Disagreements between reviewers in the process of data extraction were resolved by discussing with a third reviewer. Incomplete data will be provided by contacting corresponding authors.

### 3.7 Assessment of the methodological quality

The risk of bias for included studies will be evaluated by two reviewers using the Cochrane Collaboration's tool for assessing risk of bias.^[27]^ It includes the following seven domains: random sequence generation, allocation concealment, blinding of participants and personnel, blinding of outcome assessment, incomplete outcome data, selective reporting, and other sources of bias. Any disagreement should be solved in consultation with a third reviewer.

### 3.8 Measures of treatment effect

Weighted mean difference (WMD) or standardized mean difference (SMD) will be adopted as statistical indicators in the analysis of continuous outcomes and the relative risk (RR) will be used to assess the treatment effect for dichotomous outcomes. Ninety-five percent of the confidence intervals (CIs) will be determined in pooled estimates.

### 3.9 Dealing with missing data

We will attempt to contact authors to obtain missing data. If we cannot contact the original authors, the studies will be excluded from the data synthesis.

### 3.10 Assessment of heterogeneity

Statistical heterogeneity should be evaluated by Chi-Squared tests and *I*^2^ statistic. The results of the *I*^2^ statistic, which determine the using of fixed-effects model or random-effects model, cover unimportant heterogeneity (0–40%), moderate heterogeneity (30–60%), substantial heterogeneity (50–90%), and considerable heterogeneity (75–100%). A random-effect model or subgroup analysis should be used when there exists significant heterogeneity.

### 3.11 Data synthesis

We will run meta-analyses using the Review Manager (RevMan) V.5.3 software. If the result of heterogeneity in *I*^2^ < 40%, the fixed-effects model will be used for data synthesis and analysis; If *I*^2^ ≥40% and <75%, the random-effects model will be implied; If *I*^2^ ≥75%, it means there is considerable heterogeneity between studies. Alternatively, we will remove low-quality studies and use sensitivity analysis to investigate which study has the most significant impact on heterogeneity. If quantitative synthesis is not possible, we will make a qualitative description.

### 3.12 Subgroup analysis

If there is significant heterogeneity between the study results, we will perform a subgroup analysis to investigate differences in gender, age, outcome styles, etc.

### 3.13 Sensitivity analysis

We will perform sensitivity analyses to verify robustness of results. It includes the impact of methodological quality, study design, and sample size.

### 3.14 Grading the quality of evidence

Two reviewers will independently use the Grading of Recommendations Assessment, Development and Evaluation (GRADE), which evaluates the quality of evidence as “high,” “moderate,” “low,” or “very low,” to assess the quality of evidence.^[28]^

### 3.15 Ethics and dissemination

The study will be published in a peer-reviewed journal or relevant conference. No ethical approval is required. The results of the study will provide potential guidance in advancing the therapeutic strategy of patients with IBS.

## 4 Discussion

Depression and anxiety are the most common non-gastrointestinal symptoms in patients with IBS. The appearance of depression and anxiety greatly increases the difficulty of treatment. Although antidepressants and psychotherapy may be beneficial for functional gastrointestinal diseases (such as IBS), adverse reactions are more common in antidepressants, especially trichloroacetic acid,^[29]^ and patients have limited access to psychotherapy. Therefore, more and more healthcare providers and patients turn to traditional Chinese medicine. Acupuncture is convenient and widely used for gastrointestinal diseases. Considering that no systematic analysis exists in this regard, we aimed to perform a comprehensive systematic review and meta-analysis on the efficacy of acupuncture for depression and anxiety in IBS.

## Author contributions

**Conceptualization:** Huaiyu Li, Jing Ye.

**Data curation:** Huaiyu Li, Jiawang Jiang, Haiyi Tang.

**Formal analysis:** Yun Chen, Yuliang Zhou, Zhiying Yu.

**Methodology:** Yun Chen, Ziyi Hu, Haiyi Tang.

**Software:** Jiawang Jiang, Yuliang Zhou, Zhiying Yu.

**Supervision:** Jiawang Jiang, Jing Ye.

**Writing – original draft:** Huaiyu Li, Jiawang Jiang, Haiyi Tang.

**Writing – review & editing:** Jing Ye, Yuliang Zhou, Zhiying Yu.
